# Spatio‐temporal distribution of brain activity associated with audio‐visually congruent and incongruent speech and the McGurk Effect

**DOI:** 10.1002/brb3.407

**Published:** 2015-10-15

**Authors:** Hillel Pratt, Naomi Bleich, Nomi Mittelman

**Affiliations:** ^1^ Evoked Potentials LaboratoryTechnion ‐ Israel Institute of TechnologyHaifa32000Israel

**Keywords:** Event‐related potentials, hemispheres, multimodal Integration

## Abstract

**Introduction:**

Spatio‐temporal distributions of cortical activity to audio‐visual presentations of meaningless vowel‐consonant‐vowels and the effects of audio‐visual congruence/incongruence, with emphasis on the McGurk effect, were studied. *The McGurk effect* occurs when a clearly audible syllable with one consonant, is presented simultaneously with a visual presentation of a face articulating a syllable with a different consonant and the resulting percept is a syllable with a consonant other than the auditorily presented one.

**Methods:**

Twenty subjects listened to pairs of audio‐visually congruent or incongruent utterances and indicated whether pair members were the same or not. Source current densities of event‐related potentials to the first utterance in the pair were estimated and effects of stimulus–response combinations, brain area, hemisphere, and clarity of visual articulation were assessed.

**Results:**

Auditory cortex, superior parietal cortex, and middle temporal cortex were the most consistently involved areas across experimental conditions. Early (<200 msec) processing of the consonant was overall prominent in the left hemisphere, except right hemisphere prominence in superior parietal cortex and secondary visual cortex. Clarity of visual articulation impacted activity in secondary visual cortex and Wernicke's area. McGurk perception was associated with decreased activity in primary and secondary auditory cortices and Wernicke's area before 100 msec, increased activity around 100 msec which decreased again around 180 msec. Activity in Broca's area was unaffected by McGurk perception and was only increased to congruent audio‐visual stimuli 30–70 msec following consonant onset.

**Conclusions:**

The results suggest left hemisphere prominence in the effects of stimulus and response conditions on eight brain areas involved in dynamically distributed parallel processing of audio‐visual integration. Initially (30–70 msec) subcortical contributions to auditory cortex, superior parietal cortex, and middle temporal cortex occur. During 100–140 msec, peristriate visual influences and Wernicke's area join in the processing. Resolution of incongruent audio‐visual inputs is then attempted, and if successful, McGurk perception occurs and cortical activity in left hemisphere further increases between 170 and 260 msec.

## Introduction

### Audio‐visual integration

Simultaneous congruent inputs from a number of sensory modalities improve perception and interpretation of sensory information (Ernst 2007). Thus, the understanding of spoken words in a noisy environment can be enhanced by lip reading (e.g., Sumby and Pollack 1954; Erber 1969). When perception according to one modality is modified by concurrent information from another modality interaction among modalities occurs and it is called multimodal interaction or multimodal integration. Multimodal interaction is common (e.g., Sekuler et al. 1997; Shams et al. 2002; De Gelder 2003; Violentyev et al. 2005; Bresciani et al. 2008), fundamental to sensory perception (Calvert and Thesen 2004), and manifests in reduced reaction times (e.g., Molholm et al. 2002; Hecht et al. 2008a,b), improved orientation (Körding et al. 2007), enhanced identification of stimuli (Lovelace et al. 2003), and increased perceptual sensitivity (e.g., Frassinetti et al. 2002; Schürmann et al., 2003). Low intensity stimuli are associated with more effective multimodal interactions (the inverse effectiveness principle – Wallace et al. 1996).

The traditional view on multimodal integration has been hierarchical, with sensory processes initially acting separately followed by integration between them at later stages (described and challenged by Driver and Noesselt, 2008). This model has also been challenged by findings in the electroencephalogram (EEG) that demonstrated multimodal interactions earlier than 200 msec from stimulus onset (e.g., Eimer 2001; Fort et al. 2002a,b; Molholm et al. 2002; Teder‐Salejarvi et al. 2002; Beauchamp et al. 2004; Talsma and Woldorff 2005; Besle et al. 2009; Sella et al. 2014). These findings often related to sensory‐specific Event‐Related Potential (ERP) components such as the auditory P_50_ (Talsma and Woldorff 2005) and N_100_ (Giard and Peronnet 1999; Besle et al. 2009), the haptic N_140_ (Taylor‐Clarke et al. 2002; Forster and Eimer 2005), and the visual P_1_, N_1_ (Eimer and Driver 2000) and N_185_ (Giard and Peronnet 1999).

Specifically for speech, audio‐visual integration has been shown to depend on the subject's awareness of the stimulus being speech (Tuomainen et al. 2005). Audio‐visual integration is enhanced with training and takes place as early as the brainstem (Musacchia et al. 2007). In addition, electroencephalographic findings showed audio‐visual interaction that speeds up cortical processing of auditory signals within 100 msec of signal onset (Wildgruber et al. 2005). Interestingly, electrophysiological studies on early (<200 msec) audio‐visual speech interactions in auditory cortex indicate a probable role of sensory, attentional and task‐related factors in modulating these early interactions (Besle et al. 2009), underscoring the importance of controlling these factors in audio‐visual interaction studies.

### The McGurk–MacDonald effect

*The McGurk effect is a* specific case of audio‐visual interaction which occurs when a clearly audible syllable with one consonant, is presented simultaneously with a visual presentation of a face articulating a syllable with a different consonant and the resulting percept is a syllable with a consonant other than the auditorily presented one (McGurk and MacDonald 1976; MacDonald and McGurk 1978; Nath and Beauchamp 2012; Basu Mallick et al. 2015). This striking phenomenon is typically demonstrated by presenting an auditory/ba/with a visual/ga/, resulting in a percept of/da/. The McGurk effect is recognized as strong evidence for audio‐visual integration in speech perception (McGurk and MacDonald 1976; Fowler 1986; Massaro 1987, 1998; Summerfield 1987; Brancazio and Miller 2005) because the audio‐visual discrepant presentation results in a single speech percept incorporating phonetic information from both modalities.

The McGurk effect has been replicated with different stimuli under various conditions (Manuel et al. 1983; Green et al. 1991; Green and Gerdeman 1995; Massaro and Cohen 1996; Rosenblum and Saldana 1996; Brancazio and Miller 2005). However, it does not always occur and subject's percept may be consistent with the auditory input with no apparent effect of the visual input (Nath and Beauchamp, 2012; Basu Mallick et al. 2015). Even for an audio‐visual combination that does result in the McGurk effect, repeated presentations of the stimuli in an experiment often results in only some of the trials showing the effect (MacDonald and McGurk 1978; Massaro and Cohen 1983; Green and Norrix 1997; Brancazio 2004). Furthermore, the efficacy of a given audio‐visual combination in evoking the McGurk effect considerably varies across subjects (Brancazio et al. 1999; Carney et al. 1999; MacDonald et al. 2000; Basu Mallick et al. 2015).

Notably, absence of the McGurk effect does not necessarily result from failing to attend to the visual presentation nor does it preclude audio‐visual interaction. Thus, the voicing boundary along an auditory voice‐onset‐time continuum of a speech token has been shown to shift as a result of changing the visual speaking rate even when the McGurk effect was not evident, indicating some visual influence on the phonetic percept (Brancazio and Miller 2005).

### Acoustic and visual parameters affecting the McGurk effect

The McGurk effect is more evident with speech than with nonspeech stimuli such as clapping hands (Rosenblum and Fowler 1991), clicks (Brancazio et al. 2006), and cello sounds (Saldana and Rosenblum 1993). Moreover, click‐vowel Zulu syllables that are perceived by American English listeners as non‐speech (Best et al. 1988) yielded a McGurk effect as pronounced as with stop‐consonant‐vowel syllables (Brancazio et al. 2006). Taken together with results showing a stronger effect with consonants than with vowels (Summerfield and McGrath 1984; Massaro and Cohen 1993) these findings indicate that a rapid release of a vocal tract constriction is required for a robust McGurk effect. These results underscore the importance of acoustic properties of the auditory stimulus to the robustness of the McGurk effect (Brancazio et al. 2006).

The nature and clarity of the concomitant visual signal also affects the McGurk effect. Visual speaking rate affects the percept resulting from the audio‐visual stimulus, with faster visual displays biasing perception toward the auditory signal (Brancazio and Miller 2005), indicating decreased efficacy of the visual input and decreased robustness of the effect. Spatial quantization of the visual display, degraded from intact to increasingly coarser displays, results in decreasing efficacy of the visual input and a shift in perception toward the auditory stimulus (MacDonald et al. 2000).

### Hemispheric involvement in speech processing

Neural mechanisms underlying speech and language processing were historically localized to the left frontal lobe for speech production (Broca 1861) and to the left temporo‐parietal region for language comprehension (Wernicke 1874). More recent neuroimaging studies suggested that Broca's and Wernicke's areas were only part of a wider network including multiple supplementary areas (Pulvermüller 1996), perisylvian areas in the left hemisphere and other areas in both hemispheres, reflecting specific features of speech (Pulvermüller and Mohr 1996; Hartwigsen et al. 2010). This variability in areas was suggested to reflect a largely bilateral ventral stream which processes speech signals for comprehension, and a strongly left hemisphere dominant dorsal stream which maps acoustic speech signals to frontal lobe articulatory networks (Hickok and Poeppel 2007). A review of fMRI studies suggested localization of prelexical speech perception in bilateral superior temporal gyri. In contrast, meaningful speech perception was localized in middle and inferior temporal cortex; semantic retrieval in the left angular gyrus and pars orbitalis; whereas sentence comprehension was associated with bilateral superior temporal sulci (Price 2010).

Right hemisphere involvement has also been reported for auditory and visual speech and language tasks in functional imaging studies (Dehaene et al. 1997; Schlosser et al. 1998; Springer et al. 1999; Buchanan et al. 2000; Meyer et al. 2000; Seger et al. 2000; Chee et al. 2001; Klein et al. 2001; Bookheimer 2002). Electrophysiological studies have also indicated right hemisphere involvement in speech and language processing (Kiefer et al. 1998; Federmeier and Kutas 1999; Khateb et al. 2001; Sinai et al., 2003). The right hemisphere's role typically involves phonologic/acoustic feature extraction, prosody, and emotional expression/perception (Sinai and Pratt, 2003; Wildgruber et al. 2005). Patients who suffer from pure word deafness almost always have bilateral brain damage (Albert et al. 1981), indicating a crucial role for the right hemisphere in phonologic decoding of speech sounds. Despite this general evidence, hemispheric distribution of the specific networks involved in processing audio‐visual speech is still under debate and putative in nature (Bernstein and Liebenthal 2014).

### Brain regions associated with the McGurk effect and their activity

The trial‐to‐trial variability of the McGurk effect has been shown to depend on the brain states preceding the presentation of stimuli (Keil et al. 2012). Thus, perception of the McGurk effect was preceded by high beta activity in parietal, frontal, and temporal areas. Beta activity was pronounced in the left superior temporal gyrus (lSTG), a region involved in multimodal integration. This area was functionally associated with distributed frontal and temporal regions in trials yielding the McGurk effect. As the lSTG coupling to fronto‐parietal regions increased, so did the likelihood that multisensory information will fuse. In addition, McGurk perception was accompanied by poststimulus decreased theta‐band activity in the cuneus, precuneus, and left superior frontal gyrus while event‐related activity was more pronounced in the left middle temporal gyrus.

Neuroimaging studies suggested that in addition to Heschel's gyrus, the middle superior temporal sulcus (STS) and the middle intra‐parietal sulcus (IPS), motor speech regions of the brain, and particularly Broca's area, are also involved in resolving and fusing incongruent audio‐visual speech (Miller and d'Esposito 2005; Nath and Beauchamp 2012). The hypothesized circuit of processing, based on fMRI results, comprised initial integration of audio‐visual speech by the middle STS, followed by recruitment of the IPS, with subsequent activation of Broca's area. A high temporal resolution Event‐Related Potentials (ERP) study of the McGurk effect (Bernstein et al. 2008) showed early (<100 msec) and simultaneous activations in areas of the supramarginal and angular gyrus (SMG/AG), the intra‐parietal sulcus (IPS), the inferior frontal gyrus (Broca's area), and the dorsolateral prefrontal cortex. In addition, left hemisphere SMG/AG activation, not predicted based on the unisensory stimulus conditions, was observed in response to audio‐visual stimuli at approximately 160 to 220 msec. The STS, typically considered the site of audio‐visual speech integration (e.g., Wright et al. 2003), was neither the earliest nor most prominent activation site. However, the relatively late activity of the SMG/AG, specifically under audio‐visual conditions, was suggested as a possible audio‐visual speech integration response. In contrast to these indications of Broca's area involvement, a recent study (Matchin et al. 2014) showed that distracting the speech motor system through articulatory suppression did not result in a reduction of McGurk audio‐visual fusion. Moreover, fMRI evidence in that study did not support audio‐visual integration in Broca's area, but did find evidence for integration in the posterior temporal sulcus. Thus, previous studies on the McGurk effect are at odds regarding the involvement of Broca's area and the roles of STS and SMG/AG in the McGurk effect. A carefully controlled, high temporal resolution study of the spatio‐temporal distribution of audio‐visual integration and incongruence resolution should contribute to the resolution of conflicting results among studies using a variety of imaging methods.

### Purpose of this study

The general aim of this study was to define the distribution and time course of processing speech presented audio‐visually and to determine the effects of congruence or incongruence of the auditory and visual inputs. In addition, the clarity of the visual articulation was manipulated to study its effect on integration and incongruence resolution.

Because a variety of stimulus, attentional and task‐related factors affect audio‐visual integration (Besle et al. 2009), an ideal experiment would have subjects perform the very same task to the very same stimuli and the resulting percept would vary according to the nature of bimodal integration and incongruence resolution that took place in their brain. The McGurk effect provides a specific case of incongruence resolution in which the very same audio‐visually incongruent stimuli can result in different percepts (Nath and Beauchamp, 2012; Basu Mallick et al. 2015).

To address these aims we used auditory presentation with a synchronous visual presentation of the person articulating the same or a different utterance. The speech element studied was a consonant with a rapid release of a vocal tract constriction, as required for a robust McGurk effect. The consonant was perceived differently, depending on the audio‐visual interaction. To avoid overwhelming the brain responses to the consonant by nonspecific responses to the onset of sound (Dimitrijevic et al. 2013), the consonant was embedded between two vowels, creating a Vowel‐Consonant‐Vowel (VCV) utterance. VCVs were presented in pairs and the subjects' task was to indicate if members of the pair were same or different.

Hemispheric lateralization of speech processing has been shown to be affected by processing prosody and emotion (Albert et al. 1981; Sinai and Pratt, 2003; Wildgruber et al. 2005), spatial orientation (Sabbagh 1999; Snow 2000), lexical, grammatical, and semantic aspects of language processing (Delis et al. 1983; Faust and Chiarello 1998; Federmeier and Kutas 1999; Nieto et al. 1999; Sereno 1999; Coney and Evans 2000; Faust and Weisper 2000; Gold and Kertesz 2000; Seger et al. 2000). To avoid confounding the hemispheric lateralization of audio‐visual integration and incongruence resolution by these processes, the utterances were monotonously read, binaurally presented and meaningless. To avoid overwhelming the brain activity associated with audio‐visual integration and incongruence resolution by cognitive activity related to memory retrieval, decision making, and response selection (Pratt et al. 1989a,b), only brain responses to the first utterance in the pair were analyzed (see ERP derivation and waveform analysis). The perceptual similarity of the members of the pair could be manipulated using same or different, congruent or incongruent audio‐visual inputs.

### Hypotheses

We hypothesized that the networks involved in audio‐visual integration are dynamically distributed and parallel rather than hierarchically organized. We expected left hemisphere prominence in the activity of brain areas involved in auditory speech processing as well as in visual and association cortices, in particular with congruent audio‐visual articulation. Furthermore, we expected right hemisphere prominence in these brain areas, when careful scrutiny of unfamiliar inputs was required (Sinai and Pratt 2003), particularly with incongruent audio‐visual inputs. Similarly, we expected that once incongruence was resolved and an effective McGurk perception occurred, left hemisphere prominence, similar to clearly perceived congruent audio‐visual articulations, would be observed. We further hypothesized that as visual input clarity (but not intensity) was reduced, incongruence resolution would bias perception toward the auditory constituent of the bimodal stimulus and the effects of visual inputs on brain activity will diminish.

## Methods

### Subjects

Twenty (11 women and nine men) 22–30 years old right‐handed normal‐hearing subjects participated in the study. All subjects had normal or corrected vision, found intensities of approximately 60 dBnHL to be their most comfortable level for discriminating speech, tested negative for attention deficit (ADHD questionnaire retrieved from the DSM‐IV, 1994) and reported no history of learning disabilities. Procedures and subject recruitment in this study were approved by the Institutional Review Board for research involving human subjects and all participants signed an approved informed consent form.

### Stimuli

**Figure 1:**
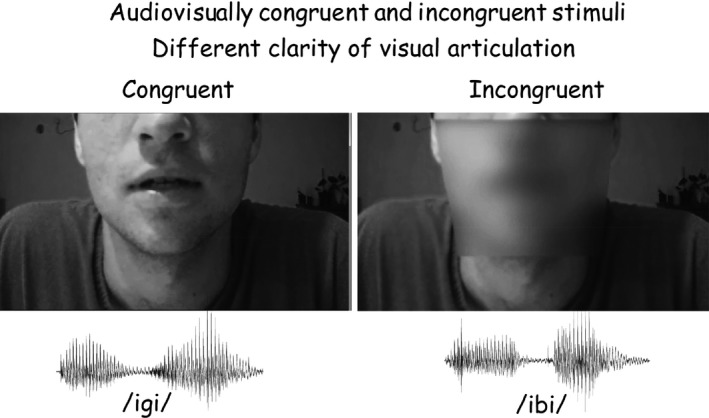
The frame of consonant articulation from the motion video display of articulation (top) and the acoustic waveforms of/igi/and/ibi/(bottom) used for the audio‐visual stimuli. Onset of the consonant was approximately 300 msec after stimulus onset, following the low amplitude waveform in the middle of the utterance. The video showed the articulation of the same VCV, producing congruent audio‐visual inputs (left), or of a different VCV (right). Clarity of the visual display was reduced by creating a 25% opacity in the mouth area (right) or by presenting a still frame rather than a motion video of the articulation.

Stimuli included three meaningless bisyllabic Vowel‐Consonant‐Vowel (VCV) sequences:/ibi/,/igi/,/idi/presented binaurally in a male voice through headphones. The bottom of Figure 1 presents the acoustic waveforms of/igi/and/ibi/. Onset of the consonant was approximately 300 msec after stimulus onset. Subjects were presented with pairs of these VCVs and a synchronous video presentation of a face articulating VCVs (Fig. 1, top). The video showed the articulation of the same VCV, producing congruent audio‐visual inputs (Fig. 1, left), or a different VCV. With an incongruent visual articulation the percept was of the auditory stimulus, or an illusion of a third consonant when the auditory stimulus was/ibi/while the video showed articulation of/igi/(Fig. 1, right). This condition of audio‐visual incongruence results in the McGurk effect, creating the perception of/idi/.

Auditory stimuli were presented binaurally at the subject's most comfortable level, typically around 60 dBnHL. The video was presented on a screen 1.5 m in front of the subject's eyes, with the face occupying an area of 36 × 27 cm on the screen, of which the area from the eyes up was occluded and lips dimensions were 6 × 12 cm. The duration of each pair of VCVs was approximately 1.5 sec with an interstimulus interval of 0.5 sec within the pair. The interval between offset of a pair and the onset of the following pair was 2 sec, such that the time interval between onset of a pair and onset of the subsequent pair was 3.5 sec. In the synchronized visual presentations of VCV articulation, two levels of visual clarity of articulation were used: (1) full clarity of the entire face (Fig. 1, top left); and (2) with a trapezoid (25.5 cm wide at the level of the nose tip and 19 cm wide at the tip of the chin, with a height of 19 cm) creating 25% opacity covering the mouth area (Fig. 1, top right). In addition, in some of the trials a display of a still face without articulation was synchronously presented with the auditory presentation, as a control for the specific role of visual articulation. Thus, there were three levels of visual articulation: full, blurred, and none (still).

Three VCV combinations within the pair were randomly presented: (1) A first stimulus comprising of an auditory/igi/and a congruent visual/igi/and a second stimulus repeating the first (/igi/in both auditory and visual presentation); (2) A first stimulus comprising of an auditory/ibi/and an incongruent visual/igi/and a second stimulus comprising of an auditory and congruent visual/igi/; and (3) A first stimulus comprising of an auditory/ibi/and visual/igi/and a second stimulus comprising of an auditory/idi/and a visual/igi/. The congruent first utterance of pair type (1) was perceived as/igi/and therefore marked as “same” by all subjects (Table 1). It was therefore considered an audio‐visually congruent utterance in all subsequent analyses. The incongruent first utterance of pair type (2) was adjusted to be perceived as its auditory constituent/ibi/or as/idi/when McGurk perception took place, but was never perceived as/igi/. It was therefore marked as “different” than the congruent/igi/of the second utterance by all subjects (Table 1). It was therefore labeled an audio‐visually incongruent utterance. The incongruent second utterance of pair type (3) was invariably perceived as its auditory constituent/idi/. In contrast, the first utterance of pair type (3), which is the typical audio‐visual stimulus resulting in McGurk perception, was perceived in some trials as/idi/(McGurk perception) and therefore marked as “same”, while in other trials it was perceived as “different” (no McGurk effect). Each visual articulation was randomly presented with full face clarity or with partial clarity (25% opacity of the mouth area) such that six stimulus pair types (3 VCV types X 2 levels of visual clarity of articulation) were presented randomly with equal probability. The control stimulus pairs with no articulation, consisting of the above 3 VCV combinations in which the synchronous visual presentation was of a still face, were presented separately in the experimental session, with equal probabilities of the three pair types. Each of the nine stimulus pair types was presented 300 times during the experimental session in a randomized sequence of pairs.

**Table 1:** Averages and standard deviations (in parentheses) of reaction times (in msec) in the stimulus–response combinations. Reaction times to the McGurk effect, to congruent and to incongruent auditory‐visual articulation are listed separately for the non‐McGurk stimulus conditions. The ‘*Same’* column lists reaction times when the subject judged both utterances in the pair to be the same, while the ‘Different’ column presents reaction times to pairs that were judged to comprise of utterances that were different from each other. McG*+* stands for trials with McGurk stimuli and an evident McGurk percept while McG*−* represents trials with McGurk stimuli in which the McGurk effect was not evident. Still indicates no articulation in the visual display (still face), *Blurred* stands for blurred mouth articulation display and Full represents full articulation in the visual display

	Same	Different
McGurk Full	1866 ± 264 (McG+)	1932 ± 143 (McG−)
McGurk Blurred	1880 ± 273 (McG+)	1878 ± 143 (McG−)
“McGurk” Still	None	1822 ± 189
Congruent Full	1831 ± 143	None
Congruent Blurred	1766 ± 128	None
Congruent Still	1703 ± 165	None
Incongruent Full	None	1950 ± 139
Incongruent Blurred	None	1980 ± 171
“Incongruent” Still	None	1851 ± 173

### Procedure

Sixty tin disk electrodes embedded in an electrode cap (Electro‐Cap International, Eaton, OH), arranged according to the extended 10–20 method of electrode placement (“10% system”, American Electroencephalographic Society, 1994) and two additional electrodes, external to the cap near the right and left mastoids (M1 and M2) were applied to the subject. The mastoidal electrodes were placed 1.5 cm above their standard positions to avoid distortion due to deviations from sphericity in the source estimation procedures. All EEG electrodes were referenced to the center of the chin. An electrode located on the forearm served as ground. Electro‐ocular activity (EOG) was recorded by an electrode placed below the left eye and two electrodes placed near the outer canthi of both eyes. In all, 62 EEG channels referenced to the middle of the chin as well as horizontal and vertical EOG channels were recorded. Electrode impedance was maintained below 5 kΩ. Potentials were band‐pass filtered on‐line (0.1 to 100 Hz, 6 dB/octave slopes), amplified (EEG: 100,000; EOG 20,000), and digitized with a 16‐bit A/D converter at 512 Hz. All data, including EEG, EOG, and stimulus and response identifiers that were synchronized with each stimulus and response onset, were stored for offline analysis. Reaction times were measured between stimulus and button press onsets.

Subjects were seated in front of a video screen with headphones on their ears, in a comfortable reclining armchair in a sound‐proof chamber and were instructed to attend to pairs of audio‐visual stimuli and indicate by an appropriate button press whether both utterances in the pair were the same, or different, regardless of possible accent differences (two alternative forced choice phonologic decision). Because a button press was required after the second stimulus in the pair across all conditions, no motor contribution to brain activity associated with the first stimulus in the pair confounded the effects of experimental conditions. During the experimental session, subjects were instructed to sit as still and quietly as possible, to fixate their gaze at the center of the video screen, and avoid blinking until after the button press of their response to each trial. Subjects were allowed breaks and refreshment at their request. The total duration of an experimental session was typically 3–4 h, including electrode application and breaks.

### ERP derivation and waveform analysis

EEG analysis and ERP derivation were conducted using the EEGLAB toolbox (Delorme and Makeig 2004; RRID:nif‐0000‐00076) and the ERPLAB package (http://erpinfo.org/erplab; RRID:nlx 155754) implemented on MATLAB 7.11.0 (MathWorks, Natick, MA). First, the continuous EEG was band‐pass filtered (0.1–24 Hz) with an Infinite Impulse Response (IIR) filter. The continuous EEG was then segmented to epochs beginning 200 msec before until 1000 msec after stimulus onset. Epochs were classified according to the 18 combinations of stimulus pair types and subject's responses (9 stimulus pair types as detailed in Subjects. X 2 response types: similar/different). Only EEG segments associated with the first utterance in the pair were further analyzed to avoid effects of memory scanning (Pratt et al. 1989a), response selection, and motor preparation and execution (Pratt et al. 1989b). DC bias (mean voltage during the 200 msec preceding stimulus onset) was removed to improve the reliability of ICA decomposition (Groppe et al. 2005).

All trials were inspected visually and trials containing activity that exceeded ±150 *μ*V were rejected. Independent Components Analysis (ICA) was applied to the recorded potentials to extract artifact‐free EEG. ICA is a computational method for separating a multidimensional signal into additive subcomponents (ICs) by assuming that the subcomponents are statistically independent of each other. ICA algorithms have been shown to successfully separate neurally generated EEG sources from artifacts of eye movement and myogenic activity (Makeig et al. 1997; Jung et al. 2000). Individual concatenated single‐trial epochs were decomposed with Infomax ICA. Artifactual ICs were identified using the ADJUST plugin (Mognon et al. 2011). ADJUST identifies four artifact classes: vertical and horizontal eye movements, blinks, and a generic artifact for anomalous activity recorded at single electrodes, typically due to loose contact. The spatial, temporal, and spectral properties of each IC were then visually inspected to confirm the classification proposed by ADJUST and to reject other ICs with stereotyped artifacts such as EKG. Then, artifact‐reduced epochs were obtained by back‐projecting the remaining nonartifactual ICA components. ERPs were obtained by separately averaging epochs associated with each of the 18 combinations of stimulus pair type x response type. Only ERPs derived from averaging at least 40 epochs were further analyzed.

**Figure 2:**
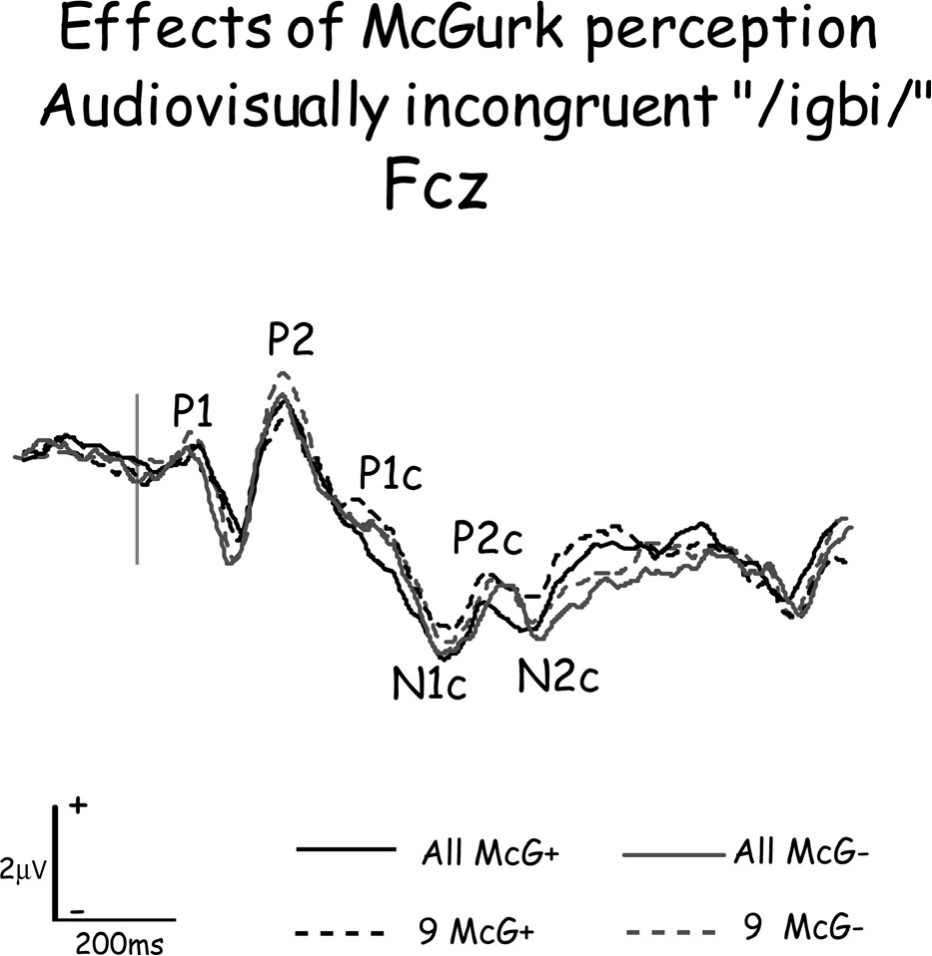
Event‐related potential waveforms recorded from FCz to the same audio‐visually incongruent stimulus when it evoked a McGurk perception (McG+) and when it failed to evoke it (McG−), when grand‐averaged from all 20 subjects or from the nine subjects that were used for analyzing the effects of McGurk perception on brain activity. The constituent peaks of the waveforms are marked by their name. The P1 to P2 complex of components were evoked by stimulus onset, while P1c to N2c are the potentials evoked by the consonant. Note the similarity of waveforms grand averaged from all subjects and from the subset of nine subjects.

Averaged responses to the first stimulus in the pair consisted of a series of peaks, time locked to different parts of the utterance: P1, N1, and P2 time locked to stimulus onset and P1c, N1c, P2c, and N2c time locked to the consonant onset (Dimitrijevic et al. 2013). Figure 2 displays ERP waveforms grand averaged from all subjects and from a subset of 9 of them (see details in Pairwise comparisons of current density distributions) recorded from FCz to the same audio‐visually incongruent stimulus (auditory/ibi/and visual/igi/) when it evoked a McGurk perception (McG+) and when it failed to evoke it (McG−), with the constituent peaks marked by their name. Grand‐average waveforms provided time frames to assist in identification and latency measurements of individual subject responses (Martin et al. 2008): P1c was defined as the positive‐going peak or inflection on the negative‐going slope following stimulus onset P2, between 280 and 370 msec following stimulus onset (~50 msec after consonant onset); N1c was defined as the negativity occurring at FCz between 400 and 440 msec (~120 msec from consonant onset); P2c was defined as the largest positive‐going peak following N1c at Fcz between 470 and 500 msec (~180 msec from consonant onset); and N2c as the following negative peak at 500–560 msec from stimulus onset (~220 msec from consonant onset).

### ERP functional imaging

Based on the distribution on the scalp of the ERPs, Standardized Low Resolution Electromagnetic Tomographic Analysis (sLORETA; Pascual‐Marqui 2002) was used to compute the cortical three‐dimensional distribution of current density. sLORETA makes no assumptions on the number of concurrently active sources. This property is particularly important in higher brain functions which involve parallel processing in multiple brain regions. Sources are suggested by minimum norm constraints and a 3‐shell head model. The solution space is restricted to cortical gray matter and hippocampus, with 6239 voxels at 5 mm spatial resolution that are registered to the Stereotaxic Atlas of the Human Brain (Talairach and Tournoux 1988). The sLORETA method is thus a properly standardized discrete, three‐dimensionally distributed, linear, minimum norm inverse solution of intracranial sources of scalp‐recorded potentials. The particular form of standardization used in sLORETA (Pascual‐Marqui 2002) results in exact localization of test point sources, yielding images of standardized current density with exact localization, albeit with low spatial resolution (i.e., neighboring neuronal sources will be highly correlated). The exact, zero‐error localization property of sLORETA has been proven (Pascual‐Marqui 2007). Furthermore, sLORETA is an improvement over previously developed tomography—LORETA, in having no localization bias even in the presence of measurement and biological noise. sLORETA has been validated in several simultaneous EEG/fMRI studies (Mobascher et al. 2009; Olbrich et al. 2009), and in an EEG localization study for epilepsy (Rullmann et al. 2009).

In this study's implementation of sLORETA, computations were made in a realistic head model (Fuchs et al. 2002) using the Montreal Neurological Institute's MNI152 template (Mazziotta et al. 2001), with the three‐dimensional solution space restricted to cortical gray matter, as determined by the probabilistic Talairach atlas (Lancaster et al. 2000). The standard electrode positions on the MNI152 scalp were used (Oostenveld and Praamstra 2001; Jurcak et al. 2007). Thus, sLORETA images represent the standardized electrical activity at each voxel in neuroanatomic Montreal Neurological Institute (MNI) space as the magnitude of the estimated current density, under the assumption that neighboring voxels should have a maximally similar electrical activity (smoothness assumption). Anatomical labels for voxels as belonging to Brodmann areas (BA) were reported using MNI space, with correction to Talairach space (Brett et al. 2002). Brain regions of interest (ROIs) were derived from the current density distribution maps. ROIs were defined as the consistently most active areas across experimental conditions.

### Statistical analyses

The estimated source current density distributions were analyzed in two ways: (1) the significance of differences in current density in specific areas at specific time intervals between pairs of experimental conditions; (2) the effects of experimental factors on the integrated activity of brain areas during time periods in which activity was consistently recorded across subjects, stimuli, and experimental conditions.

#### Pairwise comparisons of current density distributions

The significance of current density differences associated with McGurk perception were assessed by comparing current densities evoked by the same stimuli when perception was affected by the McGurk effect (subject saw/igi/and heard/ibi/and perceived/idi/) with current densities when perception was not affected by the McGurk effect (subject saw/igi/and heard/ibi/and did not perceive/idi/). For this analysis, only a subset of nine subjects that generated at least 40 trials in each condition was included. As Figure 2 demonstrates, these subjects were representative of the entire cohort.

Current density values were compared for four time periods, roughly corresponding to consonant‐evoked components P1c, N1c, P2c, and N2c, using the paired Student's *t*‐test procedure. Comparison was conducted for each period at the point in time in which the difference in current density between the two perceptual conditions (McGurk effect/no‐effect) was maximal.

#### Analysis of variance procedures

Current density values in ROIs were subjected to general linear model analysis of variance (Repeated Measures ANOVA) to test the effects of experimental factors (stimulus x response combinations, Brodmann areas and hemisphere) on current density. The Box epsilon version of the Geisser‐Greenhouse correction was applied, and any significant differences (Box epsilon probability level <0.05) were analyzed by post hoc Bonferroni multiple comparison tests.

**Figure 3:**
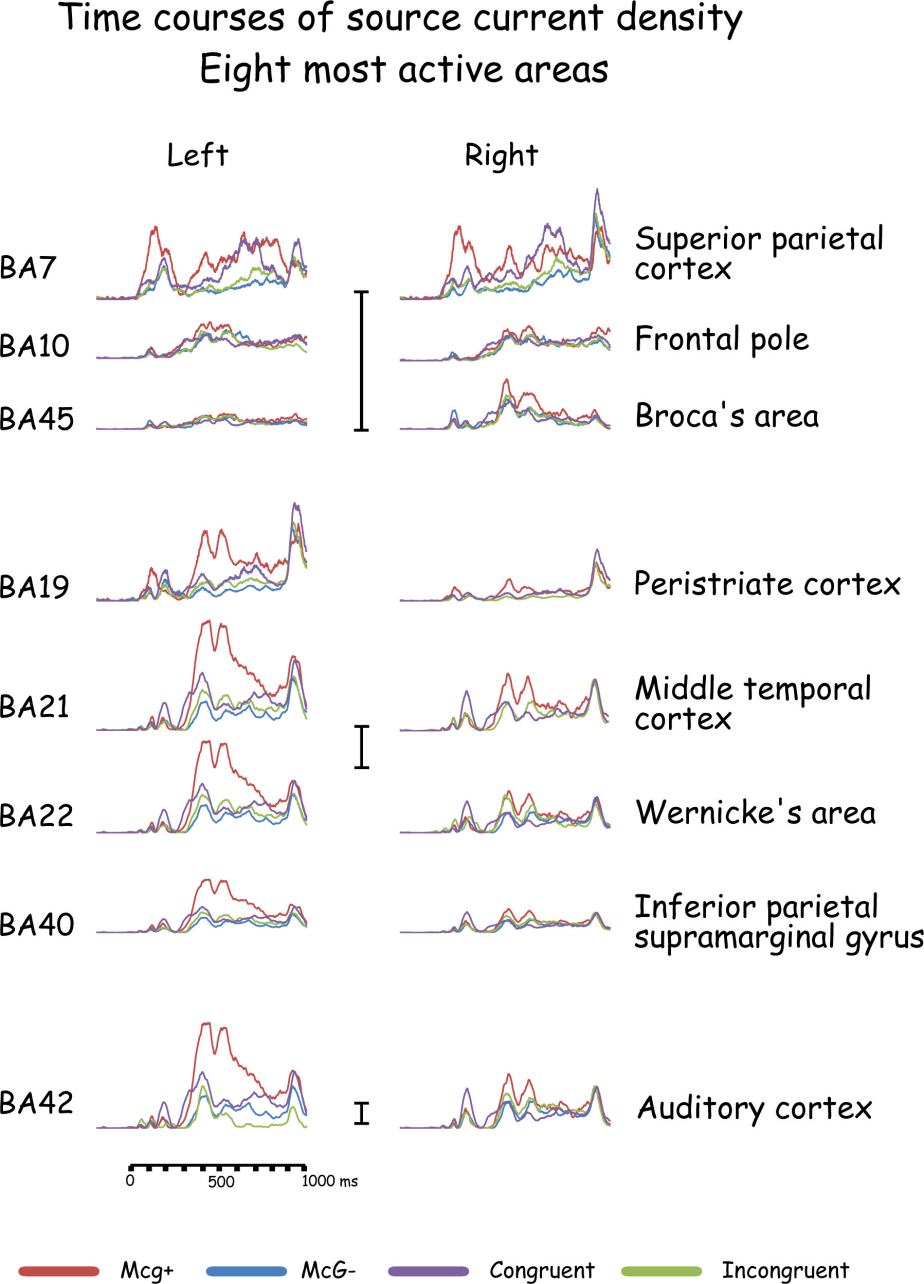
Time courses of current density in the eight most active brain areas in response to congruent and incongruent audio‐visual stimuli with full articulation. Current densities when the McGurk effect to the same incongruent stimuli was evident and when it was not are also presented. The vertical current density scales represent 1 mAmp/mm^2^.

The brain regions analyzed as ROIs were the cortical areas that were consistently found most active across experimental conditions in comparable time windows. These eight brain areas were located in the general locations of BA 7, 10, 19, 21, 22, 40, 42, and 45. For each area, source current density was integrated (current density × time, i.e., ‘area under the curve’) during 40 msec periods around the four current density peaks following consonant onset, which were consistently found to be the most active across a number of brain areas (e.g., Fig. 3). These periods roughly corresponded to the scalp‐recorded consonant‐evoked components P1c, N1c, P2c, and N2c (Fig. 2).

The significance of the McGurk effect on current density was also assessed by subjecting current densities evoked by the very same audio‐visual stimulus (visual/gi/and auditory/bi/) when the effect influenced perception (percept of/di/) and when it did not (percept different than/di/). This analysis was performed on a subset of the subjects that generated at least 40 trials in each condition. The grand‐averaged waveforms of the entire cohort and of the subset of nine subjects that had at least 40 trials for each average did not differ significantly (Fig. 2).

To assess experimental effects on current densities associated with audio‐visual integration in general, four additional ANOVAs were conducted across all experimental manipulations: (1) Effects of stimulus–response combinations, Brodmann area and hemisphere; (2) Effects of visual articulation clarity (full, blurred, and still), Brodman area and hemisphere; (3) In conditions with still visual input: effects of stimulus–response combinations, Brodmann area and hemisphere; and (4) In the stimulus–response combination in which the subject heard and saw/igi/: effects of visual input clarity (full, blurred, and still visual input), Brodman area, and hemisphere. Analyses (3) and (4) were conducted for more focused assessment of the effects of congruent visual articulation clarity (analysis 4), or absence of any visual articulation input (analysis 3). Based on the results of the initial analyses, more focused analyses were repeated separately for each Brodmann area that was found to show significant effects.

Probabilities below 0.05, after Greenhouse‐Geisser corrections for violations of sphericity (when deemed necessary) and Bonferroni (all pairwise) multiple comparison post hoc tests, were considered significant. The results section only lists main effects, interactions and post hoc analyses that were found significant.

## Results

### General overview

**Figure 4:**
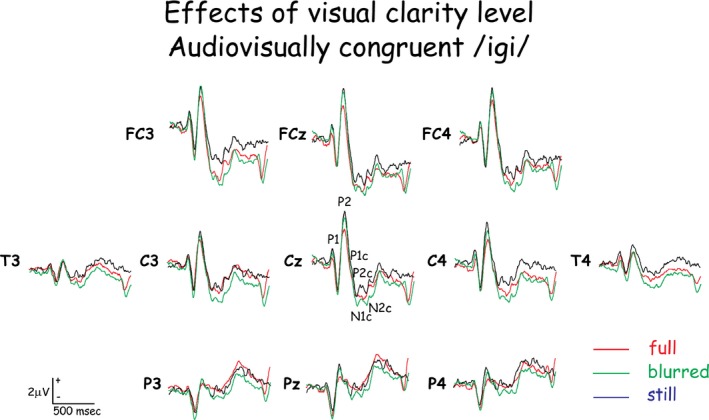
The potentials evoked on the scalp of all subjects in response to the first stimulus in the pair in response to audio‐visually congruent stimuli with three levels of visual articulation clarity. Note the similarity of waveforms in the response to stimulus onset (P1 to P2) and the differences in waveforms to the consonant (P1c to N2c).

**Figure 5:**
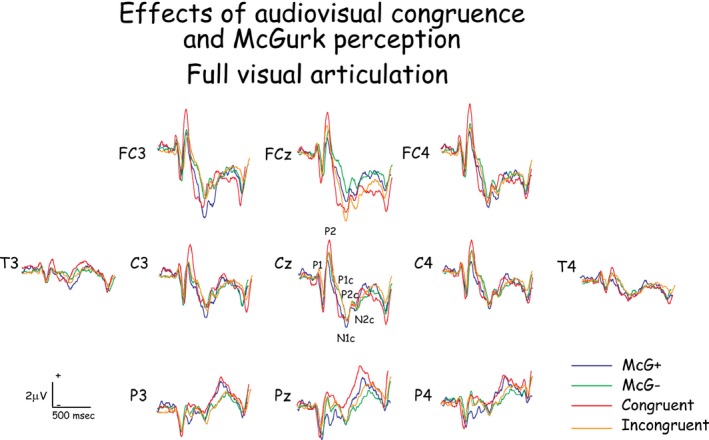
The potentials evoked on the scalp of all subjects in response to the first stimulus in the pair in response to audiovisually congruent and incongruent stimuli, with and without a McGurk perception to the incongruent stimuli.

**Figure 6:**
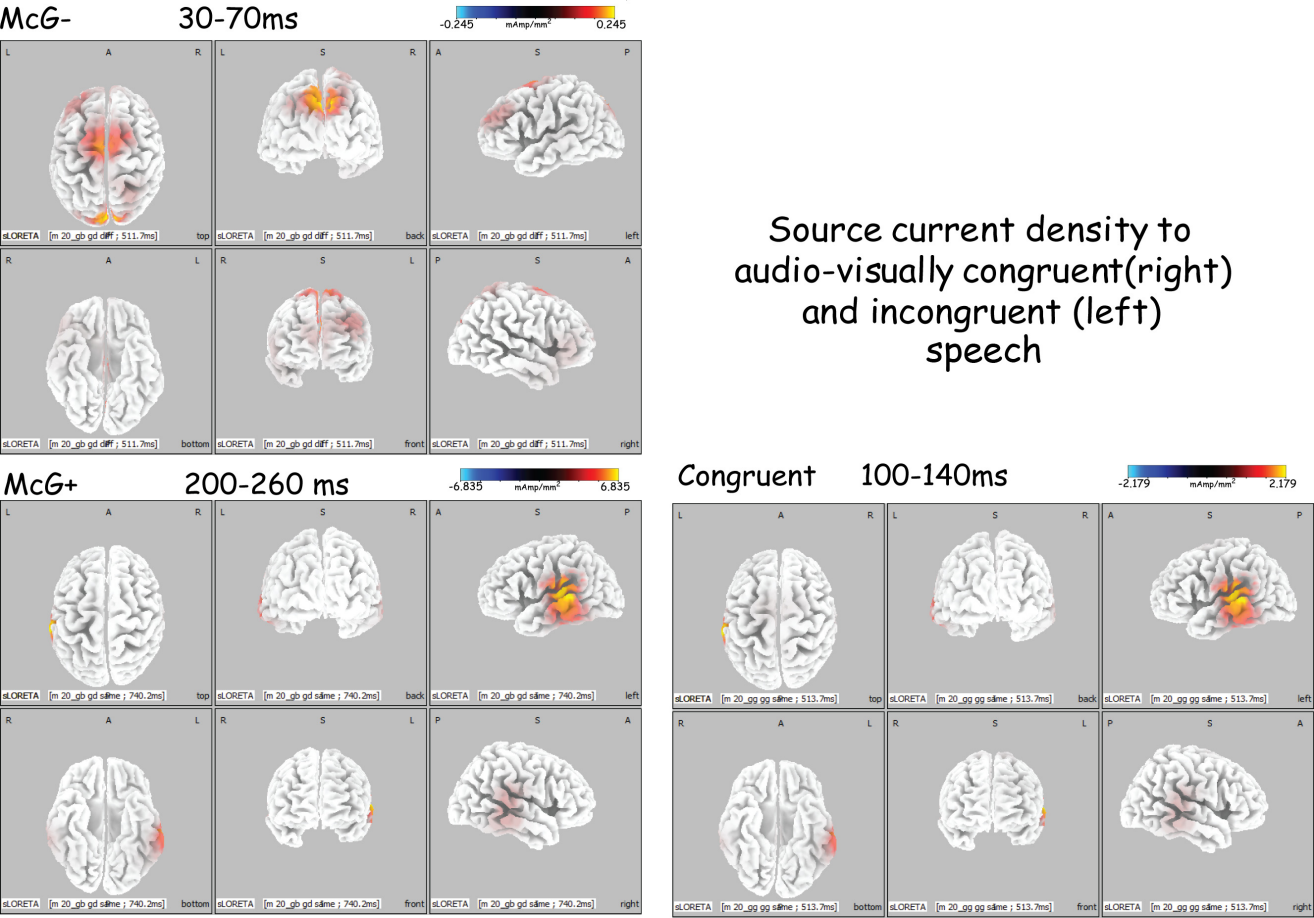
Source current density distributions to audiovisually congruent stimulus and to the same incongruent stimuli when they evoked a McGurk percept (McG+) and when they did not (McG−), at three time periods following consonant onset. Note the large number of significantly active brain areas in the initial period of 30–70 msec from consonant onset. Also note the similar distributions in response to congruent audiovisual stimuli and, a little later, to incongruent stimuli that resulted in McGurk perception.

The McGurk effect was observed in all subjects, and reaction times were shorter when the effect was observed compared to when it was not. Reaction times and the effects of experimental conditions on them are summarized in Table 1. The potentials evoked on the scalp of all subjects in response to the first stimulus in the pair included a sequence of P_1_, N_1_, P_2_, N_2_ evoked by stimulus onset, with consonant‐evoked components P1c, N1c, P2c, and N2c overlapping them. The grand‐averaged waveforms across all subjects (Figs. 4 and 5) are representative of the individual subjects' waveforms and intersubject differences are attributable to residual random noise in the recording. Source current densities of the scalp‐recorded potentials were derived (Fig. 6) and the effects of experimental conditions on intracranial activities were assessed (Tables 2 and 3) for the eight most active brain areas (Fig. 6) during four time periods defined by the time courses of intracranial activity (Fig. 3). Of these eight most active areas, all but the frontal pole (in the general location of BA10) were affected by experimental conditions. A general summary of the results relating to the study's hypotheses is provided at the end of the Results section.

**Table 2:** Significant differences in current densities to an auditory/bi/with a visual articulation input of/gi/between trials that the subject perceived as/di/(McGurk effect, *McG+*) and trials perceived otherwise (Ineffective McGurk effect, *McG−*). Comparisons were conducted on potentials to the first utterance in the pair during the 4 time periods depicted in the table. *Lt* stands for the left hemisphere, *22* represents the approximate location of Wernicke's area and *42*—the region of primary and secondary auditory cortex. Comparisons were conducted on single time point current densities using Student's *t*‐test (top) and Analysis of Variance procedures on the effect of McGurk perception on current density (bottom)

	P1c (30–70 msec)	N1c (100–140 msec)	P2c (170–200 msec)	N2c (200–260 msec)
McGurk effect	*McG− > McG+ 22Lt t*(8) = 2.786; *P* < 0.05	McG+ > McG− 22Lt *t*(8) = 1.928; *P* < 0.05		
	*McG− > McG+ 22Lt, 42Rt F*(1,6) = 7.65; *P* < 0.04	*McG+ > McG− 22Lt F*(1,6) = 9.86; *P* < 0.03	*McG− > McG+ 22Lt, 42Lt F*(1,6) = 11.71; *P* < 0.02	

**Table 3:** Significant main effects on current density and their interactions (in *Italics*) of stimulus–response combinations (*StimRes*), brain area (*BA*), hemisphere (*Hem*: Left vs. right cerebral hemisphere) and visual articulation clarity (*VisArt*), in response to first utterance in the pair during 4 time periods. *Numbers in Italics (e.g. 42*) designate the brain areas involved in the interaction by the Brodmann area roughly corresponding to them. *Rt* stands for Right, *Lt*—for Lt, *Other* represent all other conditions or brain areas, *Cong* is short for congruent auditory‐visual articulation, *Incong*—for auditory‐visual incongruence, *McG+* stands for effective McGurk effect and *McG−* represents trials in which the McGurk effect did not affect perception. *Stil* indicates no articulation, *Blur* stands for blurred mouth and *Ful* represents full articulation in the visual display

	P1c (30–70 msec)	N1c (100–140 msec)	P2c (170–200 msec)	N2c(200–260 msec)
Stimulus–response	*McG− > Other F* (6,48) = 6.89; *P* < 0.000001	*McG− > Other F*(6,48) = 10.59; *P* < 0.0000001	*McG+ > Other F*(6,108) = 21.8; *P* < 0.0000001	*McG+, − > Other F*(6,108) = 21.35; *P* < 0.000001
Brain area	*42,7,21 > Other F* (13,104) = 9.68; *P* < 0.0000001	*42,7,21,19,22 > Other F*(13,104) = 27.00; *P* < 0.0000001	*42,40,7,21,19,22 > Other F*(13,234) = 34.19; *P* < 0.0000001	*42,7,21,19,22 > Other F*(13,234) = 40.03; *P* < 0.000001
Hemisphere	*Lt > Rt F*(1,8) = 5.72; *P* < 0.02		*Lt > Rt F*(1,18) = 24.98; *P* < 0.000001	*Lt > Rt 42,40,22,21 F*(1,18) = 18.22; *P* < 0.00003
StimResXBA	See^a^ *F* (78,624) = 1.83; *P* < 0.00003			
StimResXHem	*Lt > Rt (Cong/gi/) F*(6,48) = 3.2; *P* < 0.0005			*Lt > Rt McG+ F*(6,108) = 2.51; *P* < 0.02
BAXHem	*Lt > Rt 21,22,40,42 F*(13,104) = 1.90; *P* < 0.03	*Lt > Rt; but Rt > Lt19,7 F*(13,104) = 5.66; *P* < 0.0001	*Lt > Rt 40,42,22,21; but Rt > Lt19 F*(13,234) = 2.28; *P* < 0.006	
Visual articulation	*Stil/gi/,McG− > Other F*(8,120) = 13.00; *P* < 0.00001	*Blur McG− > Other F*(8,120) = 9.42; *P* < 0.00001		
VisArtXHem	*Lt > Rt 21,22,40,42 F*(13,195) = 4.72; *P* < 0.000001			
VisArtXBA	*22 to Stil/gi/ > Other F*(104,1560) = 3.41; *P* < 0.0000001			

a *7 > Other to McG‐; 42,45 > Other to Cong/gi/*.

### Behavioral results

The McGurk effect was observed in all subjects, on average in 32% of the trials, varying across subjects between 1% and 90%. Reaction times ranged between 1770 and 1980 msec across all experimental conditions. Table 1 summarizes reaction times in the different experimental conditions, separately for subjects' judgment that the first VCV in the pair was same or different than the second. Note that in this paradigm, a judgment of “same” indicates that the first, audio‐visually incongruent stimulus was judged the same as the following congruent stimulus (i.e., the McGurk effect took place). A “different” response in this condition constitutes failure of the McGurk effect to take hold of perception. In addition to McGurk compatible stimuli with full and with blurred visual articulation, Table 1 lists reaction times in seven additional conditions of the visual display: (1) “McGurk compatible” audio‐visual combination in which the visual display was of a still face rather than articulation; three conditions of a congruent visual display: (2) with full articulation; (3) with articulation in a blurred display of the mouth; and (4) with a still display of a face without articulation; and three conditions of an incongruent visual display that does not result in the McGurk effect: (5) with full articulation; (6) with articulation in a blurred display of the mouth; and (7) with a still display of a face without articulation. Note that the McGurk effect (McG+) was elicited only with an audio‐visual stimulus that included articulation (full or blurred) but did not occur with a still visual display. Moreover, note that all congruent audio‐visual stimuli were correctly judged to be the same as the following stimulus, whereas all incongruent (auditorily different than with the McGurk condition) audio‐visual stimuli were judged to be different than the second stimulus in the pair. These results underscore the correct performance of the task by all subjects and the efficacy of the McGurk audio‐visual displays that we used to evoke the effect in some trials and to fail to do so in others. This enabled the comparison of brain activity associated with the effect or its absence in the same subjects using the very same stimuli.

Reaction times to audio‐visually incongruent stimuli that resulted in a McGurk perception (McG+) did not significantly differ (paired *t*‐test) from reaction times to the same incongruent stimuli when the McGurk effect did not take place (McG−). In contrast, reaction time to the audio‐visually incongruent stimulus that failed to evoke the effect (McG−) were significantly shorter than their counterparts in response to the congruent audio‐visual stimulus [*t*(15) = 2.6614; *P* < 0.05].

Analysis of variance of reaction times across stimulus conditions revealed a significant main effect [*F*(2,28) < 0.0002], with post hoc tests indicating that reaction times to the audio‐visual stimuli with still visual displays were significantly shorter than to all other conditions. Reaction times to the conditions with blurred visual display were shorter than to the full display, but this difference did not reach significance.

### Brain activity measures across conditions

The elctrophysiological activity varied across experimental conditions during 4 periods following stimulus onset, roughly corresponding to the four surface‐recorded peaks attributed to the consonant: (1) 280–370 msec, corresponding to surface‐recorded consonant‐evoked peak P1c; (2) 400–440 msec, corresponding to N1c; (3) 470–500 msec, during surface‐recorded P2c; and (4) 500–560 msec, during N2c. To avoid confusion with the typical latency ranges of ERP components, for simplicity's sake, all latency ranges of these components will be described relative to consonant onset by arbitrarily subtracting 300 msec from their value relative to stimulus onset. Thus, henceforth the results will be detailed for time periods following consonant onset: (1) 30–70 msec (approximately corresponding to surface‐recorded P1c); (2) 100–140 msec (N1c); (3) 170–200 msec (P2c); and 200–260 msec (N2c).

Of the eight most active brain areas, seven were consistently involved in the effects of audio‐visual experimental conditions on brain activity: (1) The superior parietal cortex, in the vicinity of Brodmann area 7 (BA7); (2) The peristriate cortex, centered in the approximate location of BA19; (3) The middle temporal cortex around BA21; (4) The superior temporal gyrus which is part of Wernicke's area, in the region of BA22; (5) The inferior parietal supramarginal gyrus, generally corresponding to BA40; (6) Auditory cortex including Heschl's gyrus and secondary auditory cortex, corresponding to the areas in the vicinity of BA41 and BA42; and (7) Inferior frontal gyrus, Broca's area, roughly corresponding to BA45. The Frontal pole, in the approximate vicinity of BA10 was among the most active areas but was not affected by experimental conditions. For brevity's sake, in the following description of the results, these seven areas will be denoted by the Brodmann areas to which they roughly correspond.

#### The McGurk effect

The significant comparisons of current density in response to the same audio‐visually incongruent stimuli when a McGurk percept was evident (McG+) and when it was not (McG−) in the four time periods analyzed is summarized in the top row (*t*‐test paired comparisons) of Table 2. The significant effects of the McGurk percept on current densities during four time periods following consonant onset are presented in bottom row of Table 2.

##### During 30–70 msec

Both analyses showed the left inferior temporal gyrus to be involved in the effect, as early as this period, with current densities higher when the McGurk effect was not evoked compared to when it was evident. The analysis of variance procedures showed an additional similar right auditory cortex involvement during this time period.

##### During 100–140 msec

Both analyses showed the left inferior temporal gyrus to be involved in the effect, but in contrast to the earlier, 30–70 msec, period current densities in this period were higher when the McGurk effect was evoked compared to when it was not.

##### During 170–200 msec

The analysis of variance procedures showed the left inferior temporal gyrus and left auditory cortex to be involved in the effect, with current densities higher when the McGurk effect was not evoked compared to when it was evident.

No significant effects of the McGurk effect on brain current density were observed during the following period (200–260 msec from consonant onset).

#### Effects of experimental manipulations and brain areas

The significant effects of experimental conditions and brain areas on current densities during four time periods following consonant onset are summarized in Table 3. More details on the specific analyses are provided in the Methods section. The striking overall finding is the large number of significant effects during the very early 30–70 msec period (9), and the appreciably smaller number of significant effects (4) during each of the later periods.

##### During 30–70 msec

Current densities were overall significantly higher in response to the incongruent audio‐visual stimulus that failed to evoke the McGurk effect compared to all other stimulus–response conditions (Stimulus–Response main effect). In response to this stimulus, current density in superior parietal cortex was significantly higher than in all other brain regions (see also in Fig. 6). In response to the audio‐visually congruent/igi/stimulus, current densities were higher in auditory cortex as well as in the inferior frontal gyrus (Broca's area)—the only significant result involving Broca's area (StimResXBA interaction). Current densities in auditory cortex, superior parietal cortex and middle temporal cortex were overall significantly higher than in the other brain areas (Brain Area main effect). Left hemisphere had significantly higher current densities than the right hemisphere (Hemisphere main effect) and in particular, left middle temporal cortex, superior temporal cortex, inferior parietal gyrus and auditory cortex (BAXHem interaction). The audio‐visually incongruent stimuli that failed to evoke the McGurk effect (with full articulation or with a still visual display) were associated with higher current densities than all other audio‐visual stimuli (Visual Articulation main effect), with higher current densities in the left hemisphere in middle temporal, superior temporal, inferior parietal and auditory cortices (VisArtXHem interaction). Current density in the superior temporal gyrus to the still visual articulation of the McGurk stimulus was higher than in all other areas in response to all other stimuli (VisArtXBA interaction).

##### During 100–140 msec

Current densities were overall significantly higher in response to the incongruent audio‐visual stimulus that failed to evoke the McGurk effect compared to all other stimulus–response conditions (Stimulus–Response main effect). Current densities in auditory cortex, superior parietal cortex, middle temporal cortex, peristriate cortex and superior temporal cortex were overall significantly higher than in the other brain areas (Brain Area main effect). Current densities in the left hemisphere were higher than in the right (see also Fig. 6), except in peristriate cortex and superior parietal cortex where current densities were higher on the right (BAXHEM interaction). In peristriate cortex trials involving visual articulation were associated with higher current densities than to still visual displays. The blurred audio‐visual stimulus that failed to evoke the McGurk effect was associated with higher current densities than the other audio‐visual stimuli (Visual Articulation main effect).

##### During 170–200 msec

Current densities were overall significantly higher in response to the incongruent audio‐visual stimulus that evoked the McGurk effect compared to all other stimulus–response conditions (Stimulus–Response main effect). Current densities in auditory cortex, inferior parietal cortex, superior parietal cortex, middle temporal cortex, peristriate cortex, and superior temporal cortex were overall significantly higher than in the other brain areas (Brain Area main effect). Left hemisphere had significantly higher current densities than the right hemisphere (Hemisphere main effect), particularly in auditory cortex, inferior parietal cortex, superior parietal cortex, and middle temporal cortex (see also Fig. 3). BA22 in the left hemisphere (Wernicke's area) had lower current density to visual articulation compared to still visual display whereas BA19 (peristriate cortex) had higher current densities on the right (BAXHem interaction), particularly to trials involving visual articulation, but not to still visual displays.

##### During 200–260 msec

Current densities were overall significantly higher in response to the incongruent audio‐visual stimulus that evoked the McGurk effect compared to all other stimulus and response combinations (Stimulus–Response main effect). Current densities in auditory cortex, superior parietal cortex, middle temporal cortex, peristriate cortex, and superior temporal cortex were overall significantly higher than in the other brain areas (Brain Area main effect). Left hemisphere had significantly higher current densities than the right hemisphere (Hemisphere main effect), particularly in auditory cortex, inferior parietal cortex, superior temporal cortex, and middle temporal cortex (see also Fig. 6). The audio‐visually incongruent stimulus that evoked a McGurk percept was associated with higher current densities in the left hemisphere (see also Fig. 3) than all other stimulus–response combinations (StimResXHem interaction).

### General summary of the results

Reaction times were significantly affected by the visual display of articulation, being significantly shorter for the still visual displays compared to the full or blurred articulation displays, and shorter to blurred visual display than to the full display, although this latter difference did not reach significance. The McGurk effect was observed in all subjects, on average in a third of the trials. Reaction times were shorter when the effect was observed compared to when it was not, but this difference did not reach statistical significance. Reaction times to the incongruent audio‐visual display that failed to evoke the McGurk effect were significantly shorter than to their congruent counterparts.

Current densities in the general vicinities of BA7 (Superior parietal lobule), BA10 (Frontal pole), BA19 (Inferior occipital gyrus, peristriate secondary visual cortex), BA21 (Middle temporal gyrus), BA22 (Superior temporal gyrus, Wernicke's area), BA40 (Inferior parietal supramarginal gyrus), BA42 (Secondary auditory cortex), and BA45 (inferior frontal gyrus, Broca's area) were higher than in other areas. Interestingly, although among the prominent areas in terms of current density, BA10 (Frontal pole) was not significantly affected by the McGurk effect nor by other experimental conditions. BA45 (inferior frontal gyrus, Broca's area) was only affected in response to congruent audio‐visual displays but not the McGurk effect itself. Early (<200 msec, around peaks P1c and N1c) processing of the consonant was overall more prominent in the left hemisphere, particularly around BA21, BA22, BA40, and BA42, and larger in the right hemisphere in the approximate locations of BA7 and BA19. In BA19 current densities were higher to trials involving visual articulation, but not to still visual displays (around peaks N1c and P2c). The effect of visual articulation level (full, blurred, still) manifested during early processing (around peak N1c) in the vicinities of BA19 and BA22. The effects of visual articulation on current densities in these areas were in opposite directions: In the vicinity of BA19 (peristriate visual cortex) in the right hemisphere, current densities were significantly higher with full visual articulation than in the other conditions, and lower than in the other conditions when a still display was presented; in contrast, in the approximate location of BA22 in the left hemisphere (Wernicke's area) current density was lower with visual articulation compared to still visual display.

Comparing current densities when the McGurk effect manifested behaviorally to when it did not, of all the brain areas compared, current densities were significantly different in the area centered at the auditory cortex and in the vicinity of Wernicke's area. Current densities in these areas were lower during very early processing (<100 msec), were higher a little later (~100 msec) and were lower again during intermediate times (~180 msec). No significant effects were observed during late processing (~230 msec). Notably, current density in BA45 (Inferior frontal gyrus, Broca's area) was not significantly affected by the McGurk effect and was only increased in response to congruent audio‐visual stimulation in the 30–70 msec period.

## Discussion

The purpose of this study was to determine if the distribution and time course of brain electrical activity associated with processing audio‐visual speech utterances are modified by congruence of the visual input (lip reading), and by the clarity of the visual articulation in general, and specifically with the McGurk effect.

The paradigm of this study included audio‐visual presentation of pairs of speech utterances, to which subjects had to respond according to whether the first and second utterance were the same or not, regardless of accent. Only potentials to the first utterance in the pair were analyzed in this modified paradigm because ERPs in the classical paradigm for the McGurk effect may include temporally overlapping memory scanning (Pratt et al. 1989a), decision making, response selection, and motor preparation activity (Pratt et al. 1989b) which may confound the specific effects of incongruence resolution.

The following discussion of our results begins with the brain areas involved in audio‐visual speech integration and with the time course of cortical activation, continues with specific changes in brain activity associated with clarity of the visual articulation of the speech utterances, followed by a discussion of specific changes in brain activity associated with the McGurk effect and incongruence resolution. Finally, these results are discussed in relation to earlier studies and suggested networks of audio‐visual integration and incongruence resolution and the hemispheric distribution of processing.

### Brain areas involved in audio‐visual speech integration

A review of fMRI studies suggested localization of prelexical speech perception in bilateral superior temporal gyri while meaningful speech perception was localized in middle and inferior temporal cortex. Semantic retrieval involved the left angular gyrus and pars orbitalis; while sentence comprehension was associated with bilateral superior temporal sulci (Price 2010). Thus, to avoid the confounding effects of speech comprehension our stimuli were meaningless, and subjects only related to their perceived meaningless phonology. Moreover, to avoid the effects of retrieval from memory (Pratt et al. 1989a,b) only potentials to the first utterance in the pair were analyzed. In addition, because only brain activity earlier than 200 msec can clearly characterize cross‐modal interactions (Besle et al. 2009; Sella et al. 2014), our analysis was limited to the initial 200 msec after consonant onset.

In this study on audio‐visual speech integration brain activity across experimental conditions (Fig. 3) was most prominent in eight areas: (1) Superior parietal lobule (BA7); (2) Frontal pole (BA10); (3) Inferior occipital gyrus, peristriate secondary visual cortex (BA19); (4) Middle temporal gyrus (BA21); (5) Superior temporal gyrus, Wernicke's area (BA22); (6) Inferior parietal supramarginal gyrus (BA40); (7) Auditory cortex (BA41,42); and (8) Inferior frontal gyrus, Broca's area (BA45). Interestingly, although among the prominent areas in terms of current density, the frontal pole (BA10) was not significantly affected by the McGurk effect nor by other experimental conditions. Due to the low spatial resolution of sLORETA, we pooled activities in primary and secondary auditory cortices as ‘auditory cortex’ (BA41,42). In contrast, we feel comfortable distinguishing current density measures in inferior parietal supramarginal gyrus (BA40) from auditory cortex (BA42) because, despite their proximity, these measures were often affected differently by the experimental conditions of this study (Tables 2 and 3).

The involvement of the frontal pole (BA10), but absence of significant effects of experimental conditions on its activity is not surprising. This area has been associated with executive functions, and in the context of this study's paradigm—with working memory (Pochon et al. 2002; Raye et al. 2002; Zhang et al. 2003), recognition (Rugg et al. 1996; Tulving et al. 1999; Ranganath et al. 2003) and recall (Zhang et al. 2003). All these functions were very much a part of the task in this study, which included comparison of the meaningless speech utterances in each pair, but these demands did not change significantly across experimental conditions.

The other brain areas, that were all significantly affected by audio‐visual congruence and clarity of visual articulation, have been implicated in processing auditory and visual aspects of this study's task. The left superior parietal cortex (BA7) has been implicated in a phonologic and semantic language task (Seghier et al. 2004), visual attention to phonemes (McDermott et al. 2003) as well as observing gestures and pantomime (Ohgami et al. 2004). Its right hemisphere counterpart has been involved in visuospatial (Kohno et al. 2006) and focused auditory attention (Hugdahl et al. 2000). The secondary visual cortex (BA19) has been implicated in sign language (Söderfeldt et al. 1997), with its left hemisphere portion involved in phonological demands (Dietz et al. 2005) and reading phonemes (Iwata 2011), while its right hemisphere portion has been implicated in visual priming (Slotnick and Schacter 2006). The middle temporal gyrus (BA21) has been reported to be involved in observing motion (Rizzolatti et al. 1996) and processing complex sounds, as well as being a part of the mirror neuron system (Arévalo et al. 2012). The inferior parietal lobule, supramarginal gyrus (BA40) has been associated with attending to phonemes in written words (McDermott et al. 2003) and in gesture imitation (Mühlau et al. 2005). Its right hemisphere constituent has been implicated in same‐different comparisons (Hirsch et al. 2001) and in observing gestures and pantomime (Ohgami et al. 2004; Mühlau et al. 2005). In addition to the involvement of primary (Heschl's gyrus) and secondary auditory cortices (BA41,42) in auditory processing, they have also been mentioned in relation to audio‐visual speech perception (Calvert and Campbell 2003; Pekkola et al. 2005) and integration (Besle et al. 2009). Thus, all the brain areas and hemispheres found to be affected by experimental conditions in this study have already been mentioned in relation to some aspects of our audio‐visual task.

### Time course of brain activation by audio‐visual speech

The high temporal resolution of the electrophysiological results can indicate the time course of brain activation by audio‐visual speech. In our results (e.g., Tables 2 and 3) this time course consists of four main periods. In the initial period (30–70 msec from consonant onset) the most active areas were Auditory cortex (BA41,42), Superior parietal cortex (BA7), and Middle temporal gyrus (BA21), all with left hemisphere prominence, particularly to the audio‐visually congruent stimuli. In response to audio‐visually congruent stimuli, Auditory cortex and Broca's area were the most active. Auditory cortex has been reported as the site of early (<200 msec) audio‐visual speech integration (Besle et al. 2009). In addition to its classic role in speech production, Broca's area (left inferior frontal cortex) has been associated with processing inputs that are congruent with pre‐existing heuristics (Tsujii et al. 2011), in line with a general suggestion of abstract internal representations that constrain the analysis of subsequent speech inputs (Wildgruber et al. 2005). Taken together these findings, in conjunction with earlier reports on the involvement of these areas, indicate that cortical audio‐visual integration begins at an early stage of processing in the auditory cortex, superior parietal cortex and the middle temporal gyrus, mostly in the left hemisphere. Audio‐visually congruent speech preferentially activated auditory cortex (BA41,42) and Broca's area (BA45). Superior temporal gyrus, Wernicke's area (BA22), also showed left hemisphere prominence compared to right, but it was not among the most active areas at this time period.

In the following time period (100–140 msec), peristriate secondary visual cortex (BA19) and Wernicke's area (BA22) join the significantly most active areas of the previous time period, and the lateralization of activity in the superior parietal cortex (BA47) and peristriate secondary visual cortex in this period is lateralized to the right hemisphere, whereas overall all other activity is lateralized to the left. Compared to the earlier time period (30–70 msec), between 100 and 140 msec from consonant onset, activity in the Superior temporal lobe and Secondary visual cortex increased in conjunction with lateralization of secondary visual and Superior parietal cortex to the right. These findings, in conjunction with previous reports on the involvement of these areas, are compatible with secondary visual (BA19) and superior parietal activity (BA7) in the right hemisphere (previously implicated in visual priming and visuospatial and focused auditory attention, respectively) contributing to increased activity in the left superior temporal lobe (BA22). During 100–140 msec left superior temporal lobe, for the first time, is among the significantly most active areas. The left superior temporal area constitutes most of Wernicke's area and, in addition to its well established role in speech and language reception, has been associated with visual phonemic attention and categorization (McDermott et al. 2003; Chou et al. 2006).

The time period between 170 and 200 msec from consonant onset exhibited the same most active brain areas as the previous period with the addition of the inferior parietal area (BA40). Overall, current densities were higher in the left hemisphere, except in secondary visual cortex (BA19), in which current densities were higher on the right, and the superior parietal cortex (BA47) that was no longer lateralized. This suggests continued activation by the right secondary visual cortex and increased activity in bilateral superior parietal cortex, which has been implicated in visual attention to phonemes (McDermott et al. 2003) and gesture imitation (Mühlau et al. 2005). The addition of the inferior parietal cortex (BA40) to the most active areas indicates, for the first time, not only visual phonemic attention (BA22) and possible involvement of mirror cells (BA21) but a motor component of imitation (see Brain networks involved in audio‐visual speech perception for further details).

During the final period analyzed, 200–260 msec from consonant onset, the inferior parietal cortex (BA40) was eliminated from the most active areas and an overall left hemisphere prominence was observed, with the exception of secondary visual cortex (BA19) and superior parietal cortex (BA7) that were no longer lateralized. Bilateral superior parietal cortex (BA7) has been associated with performance on incongruent material (Tsujii et al. 2011). This association coupled with the waning right hemisphere activity in secondary visual cortex (BA19) is compatible with a transition from attention to audio‐visual congruence/incongruence and its processing, to identifying the audio‐visual stimulus toward response selection. This time period, which parallels the surface‐recorded component N200, is compatible with the transition from stimulus evaluation to response selection in the performance of tasks (Folstein and van Petten 2008).

### Effects of the clarity of visual articulation

Earlier fMRI studies on the effects on multisensory integration of the clarity of the visual input controlled for the contribution of stimulus onset and offset cues and gross visual motion that are not specific to place of articulation (Callan et al. 2004). Similarly, our study controlled for these factors by using the very same audio‐visual inputs in which the only manipulation was blurring of the mouth area, keeping gross movements and onset/offset the same. By analyzing the effects of three levels of visual articulation: Full, blurred and still, the effects on brain activity that were found significant could be attributed to visual articulation. Because our study used functional imaging with a higher temporal resolution than fMRI, we could add information on the sequence of activation of the structures implicated by fMRI studies. Effects of the clarity of visual articulation were significant only during the initial periods of 30–70 msec and 100–140 msec from consonant onset. The main effects were overall increased activation to blurred visual articulation in response to: (1) audio‐visually incongruent stimuli that failed to evoke McGurk perception; (2) still congruent audio‐visual stimuli, specifically in the superior temporal cortex (BA22). Taken together, these findings indicate that only early (30–140 msec from consonant onset) brain activity is affected when visual articulation is degraded, manifesting in increased activity in response to the ambiguous stimuli. This results in a percept corresponding to the auditory component of the stimulus. Successful resolution of the incongruence is not accompanied by increased activity at this time, but does manifest in increased activity in later periods (170–260 msec) associated with a McGurk perception.

### Brain areas specifically involved in incongruence resolution and McGurk perception

There is disagreement on the relative contributions of stimulus parameters, brain processing and brain states in the elicitation of the McGurk effect to incongruent audio‐visual stimuli. On the one hand there is evidence that acoustic properties of the auditory stimulus and robustness of the visual input (MacDonald et al. 2000; Brancazio and Miller 2005), rather than speech‐specific brain processing, contribute to the magnitude of the McGurk effect. On the other hand, developmental age (5–19 years) of listeners significantly correlated with the number of trials in which the McGurk effect was evident (Trembley et al. 2007), linguistic experience of the listener affected the robustness of the McGurk effect (Sekiyama 1997; Sekyama and Tohkura, 1991) and EEG changes were found to have a predictive value in determining whether the effect will take place (Keil et al. 2012). All this evidence points to a significant role of brain state and processing in the McGurk effect. The results of this study, estimating sources and magnitude of activity in the brain, coupled with changes in the clarity of visual articulation and its congruence with the auditory stimulus allowed resolving this disagreement.

The results of our study show that brain activity is clearly a factor in determining whether the McGurk effect is elicited or not. At the same time the results confirm that the clarity of visual articulation affects the outcome of processing incongruent audio‐visual stimuli that evoke the effect. In the analyses on the specific effects of the McGurk effect on brain activity (Table 2), both analyses showed the left inferior temporal gyrus (BA22) to be involved in the effect, as early as 30–70 msec from consonant onset. Current densities in this area were higher when the McGurk effect was not evoked compared to when it was evident. In addition, a similar right auditory cortex (BA42) involvement during this time period was found. The left inferior temporal gyrus (BA22) continued to be involved in the effect during 100–140 msec, but in contrast to the earlier period current densities in this period were higher when the McGurk effect was evoked compared to when it was not. Finally, during 170–200 msec from consonant onset these areas were involved, and their current densities to the very same stimuli were higher when the McGurk effect was not evoked compared to when it was evident. Clearly, these results show temporal lobe areas to be involved in the McGurk effect during early processing (<200 msec) of incongruent audio‐visual stimuli. Moreover, these results on brain activity to incongruent audio‐visual stimuli show that differences in brain activity during processing of the very same incongruent audio‐visual stimuli in the same subjects, result in different perceptual outcomes.

An earlier study found shortened electroencephalographic latencies in response to congruent audio‐visual speech compared to auditory inputs alone within 100 msec from signal onset (Wildgruber et al. 2005). However, this study did not manipulate audio‐visual congruence. Another study of the McGurk effect and audio‐visual congruence effects on the surface‐recorded auditory N1 and P2 found effects that began only 200 msec after congruence or incongruence became apparent (Baart et al. 2014), much later than the earliest intracranial effects estimated in our study (30–70 msec).

The results also show that the robustness of visual articulation affects early brain activity associated with congruence of the audio‐visual stimulus and, specifically, with the McGurk effect. During the very early period (30–70 msec), overall brain current densities, in particular in the superior temporal cortex (BA22), in response to congruent but visually still audio‐visual stimulus and to incongruent stimuli that failed to elicit the McGurk percept were higher than to other stimulus conditions, particularly in the superior parietal lobule (BA7). In addition, in the following period (100–140 msec) incongruent audio‐visual stimuli with blurred articulation that failed to elicit McGurk perception were associated with higher current densities than other stimuli. These findings on the effect of clarity of visual articulation on early processing of audio‐visual stimuli can be summarized as increased activity in response to degraded visual articulation and failure to elicit the McGurk perception.

The particular brain areas with such early increases in activity (BA7 and BA22) have been implicated in processing phonology and semantics (Seghier et al. 2004), visual attention to phonemes (McDermott et al. 2003), observing gestures and pantomime (Ohgami et al. 2004), visuospatial (Kohno et al. 2006) and focused auditory attention (Hugdahl et al. 2000) as well as phoneme and auditory language processing (Söderfeldt et al. 1997; Tervaniemi et al. 2000; Ahmad et al. 2003). However, when incongruent audio‐visual stimuli did evoke the McGurk effect, this was associated with higher current densities during the later time periods (170–260 msec) and during the last period analyzed in this study (200–260 msec)—with more prominence in the left hemisphere. Taken together these effects can be summarized as early increased resource allocation in processing the degraded audio‐visual stimuli, more often than not—resulting in perception of the auditory constituent. When early processing for audio‐visual integration was successful, later activities were higher, particularly in the left hemisphere.

### Brain networks involved in audio‐visual speech perception

The sequence of activation of brain areas during the initial quarter of a second from consonant onset suggests the following network subserving audio‐visual integration for speech recognition: initial audio‐visual integration takes place 30–70 msec from consonant onset in auditory cortex, superior parietal cortex and the middle temporal gyrus, mostly in the left hemisphere. Interestingly, most of the significant effects of the clarity of visual articulation occurred during this period, even though no cortical visual areas were significantly affected. In the absence of significant effects of stimulus and response conditions on visual cortical areas at this time, integration and the effects of visual articulation may involve subcortical audio‐visual inputs to these areas, possibly from the superior colliculus (Meredith and Stein 1983; Calvert et al. 2001; Fairhall and Macaluso 2009). Cortical visual inputs are only observable beginning at 100 msec, in secondary visual cortex (BA19) and Superior parietal cortex (BA7) in conjunction with increased activation of the Superior temporal cortex (BA22). Superior temporal cortex, most often associated with audio‐visual speech processing (Wright et al. 2003; Miller and d'Esposito 2005; Bernstein et al. 2008; Smith et al. 2013), may thus be activated by cortico‐cortical connections from the other affected areas. Whereas possible involvement of the mirror cell system (BA21) is evident from the very early period following consonant onset, possible motor involvement (gesture imitation, Mühlau et al. 2005) across all experimental conditions was only indicated during a limited period between 170 and 200 msec in the inferior parietal cortex (BA40). An fMRI study on brain regions involved with perceptual enhancement by visual speech information also found activity in brain regions that are involved with planning and execution of speech production in response to visual speech presented with degraded or absent auditory stimulation. This was suggested to be consistent with speech perception facilitation by internally simulating the intended speech act of the observed speaker (Callan et al. 2003). Our results add the timeframe during which this process may take place. The involvement of motor planning in the network serving phonetic interpretation has been suggested by other studies as well (e.g., Skipper et al. 2007).

Notably in our results, Broca's area involvement was limited to the very early period, and then only to audio‐visually congruent stimuli (matching pre‐existing heuristics of congruent audio‐visual speech). This limited early involvement of Broca's area may resolve the disagreement on its involvement in the McGurk effect (Matchin et al. 2014 vs. Miller and d'Esposito 2005). In an fMRI study on audio‐visual integration of speech, regions consistently involved in perceptual fusion *per se* included Heschl's gyrus, superior temporal sulcus, middle intraparietal sulcus, and inferior frontal gyrus (Miller and d'Esposito 2005). In contrast, another fMRI study incorporating articulatory suppression indicated that the motor system was not involved in audio‐visual integration and specifically, that audio‐visual speech processing did not involve Broca's area (Matchin et al. 2014). Our results, as well as those of another ERP study (Bernstein and Miller, 2008), did find activation of Broca's area (to audio‐visually congruent stimuli) as well as supramarginal cortex activation, but for short periods (30–70 msec and 170–200 msec, respectively) that may have been too short or marginal for the temporal resolution of fMRI.

An ERP study on audio‐visual integration (Bernstein et al. 2008) challenged the ability of fMRI with its low temporal resolution to study spatio‐temporal distributions of processing and found a dynamically distributed network, including simultaneous patterns of activations in areas that had been hypothesized (Miller and d'Esposito 2005) to be activated sequentially. Our results support parallel involvement of numerous areas, including auditory cortex (BA42), superior parietal cortex (BA7), and middle temporal gyrus (BA21) which were active in parallel throughout the initial 260 msec from consonant onset, as well as peristriate cortex (BA19) and Wernicke's area (BA22) which were active in parallel to the others, beginning 100 msec from consonant onset. Thus, our results support dynamically distributed parallel processing of audio‐visual integration, with initial subcortical inputs followed by cortical visual influences and their effect on Wernicke's area. We also confirm that by 260 msec, audio‐visual integration is completed and a transition takes place from attention to audio‐visual congruence/incongruence to identifying the audio‐visual stimulus toward response selection.

### Hemispheric prominence

Contrary to our expectations of right hemisphere prominence with incongruent inputs, there was an overwhelming left hemisphere prominence in the effects of stimulus and response conditions on the brain activity associated with audio‐visual integration, regardless of congruence. The only exceptions of right hemisphere prominence were observed, for limited times only. Initially (30–70 msec), right auditory cortex was more active to stimuli that failed to elicit the McGurk percept (Table 2, bottom). Notably, the left hemisphere's Wernicke's area was also significantly more active during that period to the same audio‐visually incongruent stimuli. Later (100–200 msec), right hemisphere prominence was observed only in areas associated with visual and visuospatial attention as well as focused auditory attention (BA 19 and BA7, Table 3, BAXHem). These findings suggest that the stimuli we used were identified as speech very early in processing (30–70 msec), based on the auditory input, and were processed as such. Later (100–140 msec) attempts to resolve audio‐visual incongruence focused on the visual input and its relation to the auditory input. When audio‐visual incongruence was resolved, left hemisphere prominence was evident from 200 msec on, and McGurk perception of the incongruent audio‐visual stimulus took hold.

## Conclusions

The results of this study suggest eight brain areas involved in dynamically distributed parallel processing of audio‐visual integration. The results show the temporal sequence of events with initial (30–70 msec) subcortical contributions to auditory cortex, superior parietal cortex and middle temporal cortex. Somewhat later (100–140 msec), cortical visual influences and their effect on Wernicke's area join in. We also suggest that resolution of incongruent audio‐visual inputs is attempted between 100 and 140 msec from consonant onset, and if successful, it is associated with the McGurk perception and increased activity between 170 and 260. By that time a transition takes place from attention and resolution of audio‐visual congruence/incongruence to the identification of the audio‐visual stimulus toward response selection.

## Conflict of Interest

None declared.
